# *KCNJ16*-depleted kidney organoids recapitulate tubulopathy and lipid recovery upon statins treatment

**DOI:** 10.1186/s13287-024-03881-3

**Published:** 2024-08-26

**Authors:** E. Sendino Garví, G. J. J. van Slobbe, E. A. Zaal, J. H. F. de Baaij, J. G. Hoenderop, R. Masereeuw, M. J. Janssen, A. M. van Genderen

**Affiliations:** 1 Division of Pharmacology, Utrecht Institute for Pharmaceutical Sciences, Utrecht University, Universiteitsweg 99, 3584 CG Utrecht, The Netherlands; 2 Division of Cell Biology, Metabolism and Cancer, Department Biomolecular Health Sciences, Faculty of Veterinary Medicine, Utrecht University, Utrecht, Netherlands; 3 Department of Medical BioSciences, Radboud University Medical Center, Nijmegen, Netherlands

**Keywords:** KCNJ16, Kir5.1, Kidney organoids, Tubulopathy, iPSCs, Salt wasting, Statins treatment, Genetic kidney disease

## Abstract

**Background:**

The *KCNJ16* gene has been associated with a novel kidney tubulopathy phenotype, viz*.* disturbed acid–base homeostasis, hypokalemia and altered renal salt transport. *KCNJ16* encodes for Kir5.1, which together with Kir4.1 constitutes a potassium channel located at kidney tubular cell basolateral membranes. Preclinical studies provided mechanistic links between Kir5.1 and tubulopathy, however, the disease pathology remains poorly understood. Here, we aimed at generating and characterizing a novel advanced in vitro human kidney model that recapitulates the disease phenotype to investigate further the pathophysiological mechanisms underlying the tubulopathy and potential therapeutic interventions.

**Methods:**

We used CRISPR/Cas9 to generate *KCNJ16* mutant (*KCNJ16*^+/−^ and *KCNJ16*^−/−^) cell lines from healthy human induced pluripotent stem cells (iPSC) *KCNJ16* control (*KCNJ16*^*WT*^*)*. The iPSCs were differentiated following an optimized protocol into kidney organoids in an air–liquid interface.

**Results:**

*KCNJ16*-depleted kidney organoids showed transcriptomic and potential functional impairment of key voltage-dependent electrolyte and water-balance transporters. We observed cysts formation, lipid droplet accumulation and fibrosis upon Kir5.1 function loss. Furthermore, a large scale, glutamine tracer flux metabolomics analysis demonstrated that *KCNJ16*^−/−^ organoids display TCA cycle and lipid metabolism impairments. Drug screening revealed that treatment with statins, particularly the combination of simvastatin and C75, prevented lipid droplet accumulation and collagen-I deposition in *KCNJ16*^−/−^ kidney organoids.

**Conclusions:**

Mature kidney organoids represent a relevant in vitro model for investigating the function of Kir5.1. We discovered novel molecular targets for this genetic tubulopathy and identified statins as a potential therapeutic strategy for *KCNJ16* defects in the kidney.

**Supplementary Information:**

The online version contains supplementary material available at 10.1186/s13287-024-03881-3.

## Background

Renal tubulopathies are a broad cluster of individual rare diseases that are mostly characterized by disruptions in cell homeostasis due to genetic defects in key renal transport proteins [1]. The *KCNJ16* gene has recently been associated with a novel kidney tubulopathy phenotype. Patients carrying biallelic loss-of-function mutations in *KCNJ16* exhibit disturbed acid–base homeostasis, severe hypokalemia, polyuria, and salt wasting [2]. *KCNJ16* encodes for the inward rectifier potassium channel Kir5.1, which forms functional heterodimers with Kir4.2 (encoded by *KCNJ15*) and Kir4.1 (encoded by *KCNJ10*) [3]. These proteins function as potassium channels that regulate the basolateral membrane potential of several kidney epithelial cells [4, 5]. Altered membrane potential through dysfunctions of Kir4.1/Kir5.1 or Kir4.2/Kir5.1 heteromeric channels leads to dysregulation of several membrane potential-dependent transport processes and protein activities [6, 7], resulting in a tubulopathy phenotype as observed in patients with *KCNJ16* loss-of-function [2, 3].

In the proximal tubule (PT), Kir4.2/Kir5.1 channels regulate bicarbonate reabsorption from the PT cells into circulation via a direct feedback loop between the membrane potential in response to changes in intracellular pH, and the activation/inhibition of the Na-bicarbonate cotransporter 1 (NBC1; *SLC4A4*) [8–10]. Dysfunction of Kir4.2/Kir5.1 leads to metabolic acidosis, accompanied by an increase in intracellular pH, membrane depolarization and reduced NBC1 activity in the PT [9, 11]. In the distal convoluted tubule (DCT), Kir5.1 acts both as a potassium transporter as well as an extracellular potassium sensor involved in the potassium conductance of the basolateral membrane of the cells by adjusting the intracellular chloride concentration [12, 13]. Furthermore, Kir4.1/Kir5.1 channels showed to be involved in sodium reabsorption (via the epithelial Na^+^ channel (ENaC) [14]) and potassium secretion (facilitated by the outer medullary potassium channel (ROMK) [15]) in the collecting duct (CD).

Previous evidence suggests that defects in potassium transporters such as *KCNJ10* and *KCNJ15* is associated with a kidney phenotype, there is no precedent for *KCNJ16* defects alone causing such a phenotype except for the recently published work by Schlingmann et al*.* [2]. Furthermore, the other studies describing the involvement of *KCNJ10* and *KCNJ15* in kidney disease are mostly based on rodent models [16–20]. This substantiates the need for translational models to better understand the mechanism of disease in humans and to study therapeutic interventions. Human kidney organoids are considered advanced in vitro models that could potentially bridge the knowledge gap. Recent advances in this field demonstrated that human induced pluripotent stem cell (iPSC)-derived kidney organoids form highly organized, polarized, 3D structures containing most cell types of the nephron, and respond to environmental cues [21–24]. Moreover, iPSC-derived kidney organoids combined with CRISPR/Cas9 technology have shown to be a powerful tool to study genetic kidney disorders by recapitulating complex pathological markers/processes such as cyst formation in polycystic disease [25–27]. Therefore, the generation of an advanced, human-derived in vitro model that recapitulates aspects of the phenotype offers a tool to further study the disease and to create a personalized, pharmacotherapeutic platform for intervention studies.

In this study, we generated *KCNJ16* knock-out human iPSC-derived kidney organoids to provide an advanced and more human-translatable model to study the underlying pathophysiological mechanisms of Kir5.1 dysfunction. Additionally, we used this system to further unravel potential disease-causing mechanisms and to evaluate the effect of several drug compounds as novel therapeutic options for kidney disease caused by defects in *KCNJ16*.

## Methods

### Antibodies and reagents

All reagents were obtained from Sigma Aldrich (Zwijndrecht, The Netherlands) unless specified otherwise. The primary antibodies used in this study were sheep anti-nephrin (#AF4269-SP, R&D systems, Abingdon, United Kingdom) diluted 1:300, mouse anti-E-Cadherin (#610181, BD Biosciences, Mississauga, ON, Canada) diluted 1:300, rabbit anti-GATA-3 (#5852S, Cell Signaling Technology, Leiden, The Netherlands) diluted 1:300, biotinylated-LTL (#B-1325, Brunschwig Chemie, Amsterdam, The Netherlands) diluted 1:300, rabbit anti-Kir5.1 (#HPA059563, Bio-Connect B.V., Huissen, The Netherlands) diluted 1:300, mouse anti-ZO-1 (#610966, BD Biosciences, Vianen, The Netherlands) diluted 1:200, sheep anti-fibronectin (#AF1918, R&D systems) diluted 1:400, rabbit anti-collagen-I (#ab34719, Abcam, Amsterdam, The Netherlands) diluted 1:300. Primary antibodies were detected using Alexa-647 Donkey anti-sheep (#A-21206) diluted 1:400, Alexa-568, Donkey anti-rabbit (#A10042) diluted 1:400, Alexa-488 Donkey anti-rabbit (#A-21206) diluted 1:400, Alexa-488 Donkey anti-mouse (#A21202) diluted 1:400, Alexa-405 streptavidin conjugate (#S32351) diluted 1:400, all purchased from ThermoFisher Scientific, Breda, The Netherlands. To measure the intracellular pH, the pHrodo™ Red P35372 probe (Thermofisher) was used.

### Cell lines and cell culture

iPSCs were obtained via a material transfer agreement from The Stem Cell Technology and differentiation Center (SCTC) at the Radboudumc, Nijmegen [28]. The iPSCs were cultured in Essential 8 (E8) medium containing E8 supplement and 100 μg/mL Penicillin–Streptomycin (E8 complete medium, all from GIBCO, Life Technologies, Paisley, United Kingdom) in well plates that were coated with 1% Geltrex (ThermoFischer Scientific). Cells were cultured at 37 °C in a humified atmosphere in the presence of 5% CO2. When sufficient confluency was reached, cells were washed twice with 1 × Dulbecco’s phosphate buffered saline (PBS, ThermoFisher Scientific) and passed into colonies using 0.5 mM EDTA (ThermoFischer Scientific) for 4 min at RT. iPSC colonies were collected by addition of E8 complete medium after which colonies were transferred to fresh geltrex coated well plates. To obtain single cells, confluent iPSCs were washed twice with 1 × PBS and dissociated into single cells using TrypLE Select Enzyme (ThermoFisher) for 2 min at 37 °C. Dissociation was stopped by addition of E8 complete medium, after which cells were pelleted by spinning down at 400rcf for 5 min. The *KCNJ16*^+/−^ line harbors a heterozygous frameshift mutation in the exon 5 of the *KCNJ16* gene while the *KCNJ16*^*−/−*^ line harbors a compound heterozygous frameshift mutation in both alleles of the exon 5 of the *KCNJ16* gene (Figure S1).

### Nucleofection of iPSCs

The *KCNJ16* gene was mutated in healthy iPSCs (KCNJ16^WT^) using CRISPR/Cas9 combined with nucleofection. Duplex RNA was generated by hybridizing crRNA containing the *KCNJ16* guiding sequence (‘5- ‘3 UGAUGCAACUUAAGAUGGAC, IDT, Leuven, Belgium) with fluorescently labeled tracRNA (ATTO 488 and 550, both from IDT) for 5 min at 95 °C in a PCR machine (PCR ThermoCycler, Biorad). Next, ribonucleoproteins (RNPs) were generated by addition of Cas9 (Sigma Aldrich) to the gRNA followed by a 20-min incubation at room temperature. Finally, electroporation Enhancer (IDT) was added, the final reaction mixture consisted of 1.5 μM gRNA, 750 nM Cas9 and 0.05 ng/μL electroporation enhancer. iPSCs were pre-incubated with 10 μM revitacell (GIBCO) 1 h prior to nucleofection. Approximately 1.0*10^6^ iPSCs were gathered as single cells as described under ‘cell lines and cell culture’ and resuspended in 100 μL NucleofectorTM Solution V (Lonza, Basel, Switzerland) and added to the reaction mixture, after which the mixture was transferred to an electroporation cuvette and cells were electroporated in a 2b-Nucleofector (Lonza). Finally, cells were seeded in geltrex coated well-plates containing E8 complete medium supplemented with 10 μM revitacell.

### Cell sorting with fluorescence-activated cell sorting (FACS)

Cells were dissociated into single cells 16–24 h after nucleofection, as described in under ‘cell lines and cell culture’. Collected cells were resuspended in E8 complete medium and transferred through a 40 μm cell strainer (Corning Incorporated, NY, USA) to obtain single cells. Next, cells were sorted into single cells at the Flow Cytometry and Cell Sorting Facility at the Faculty of Veterinary Medicine (Utrecht, the Netherlands). Cells that showed fluorescence in the 488 or 550 (depending on the used tracRNA) spectra were selected and seeded as single cell per well in a Geltrex coated 96-well plate containing E8 completed medium supplemented with Clone-R (STEMCELL Technologies, Vancouver, Canada). Plates were spun down at 400rcf for 1 min to increase single cell attachment and were expanded at 37 °C in a humified atmosphere in the presence of 5% v/v CO_2_ until colonies were formed that could be used to generate a culturable cell line.

### DNA extraction and PCR

iPSCs were gathered as single cells as described under ‘cell lines and cell culture’. Cell pellets were washed once with 1 × PBS after which DNA was isolated using the QIAamp DNA Mini Kit (Qiagen, Venlo, The Netherlands) according to manufacturer’s protocol. Next, the *KCNJ16* gene was amplified with 1 × Q5 Hot Start High-Fidelity master mix (New England Biolabs, Ipswich, United Kingdom), 0.5 μM forward primer (‘5- ‘3 CTACCCGCCAGAGCACATTAT, ThermoFischer) 0.5 μM reverse primer (‘5- ‘3 TCTCGAACTGGTGGTGCTTT, ThermoFischer) and 100 ng template DNA. The resulting PCR product was purified using the QIAquick PCR Purification Kit (Qiagen) according to manufacturer’s protocol. Finally, the PCR products were sent for sanger sequencing to Macrogen Europe (Amsterdam, The Netherlands). CRISPR/Cas9-induced mutations were detected using TIDE web tool (https://tide.nki.nl/).

### Differentiation of iPSCs into kidney organoids

iPSCs were gathered as described under ‘cell lines and cell culture’. and were seeded at a density of (500,000 cells/well) in 1% w/v Geltrex coated 6-well plates in E8 containing 10 μM Revitacell for maximum 24 h. Differentiation into kidney organoids was performed by following the protocol recently described by Jansen, J et al. [29]. Culture medium was changed to Essential 6 (E6, GIBCO) medium supplemented with 6 μM CHIR99021 (R&D systems). Ureteric bud progenitor cells were generated by exposing the cells to the indicated CHIR99021 concentration for 4 days in E6 medium, after which the cells were exposed for 3 days to 200 ng/ml FGF-9 (R&D systems) and 1μg/mL heparin (Sigma Aldrich) in E6 medium. Metanephric mesenchymal cells were generated by exposing the cells to 6 µM CHIR99021 in E6 medium for 5 days and exposing for 2 days to 200 ng/ml FGF-9 and 1 μg/mL heparin. After differentiation (day 7) into ureteric bud progenitor and metanephric mesenchyme cells, organoids were generated. Cells were washed once using PBS and dissociated into single cells using 0.05% w/v trypsin for 3 min at 37 °C. Dissociation was stopped by addition of 2 volumes of DMEM-F12 with 10% FCS (GIBCO) and cells were counted. Per organoid, 100,000 Ureteric bud progenitor cells and 200,000 mesenchymal cells were mixed in E6 medium and spun down three times at 300rcf in 1.5 mL Eppendorf tubes. The obtained cell pellets were transferred using sterile wide bored pipets onto a Transwell filter (Corning or CellQart) and exposed for 1 h to 5 μM CHIR99021 in E6 after which the medium was changed to E6 containing FGF9 and Heparin. Organoids were cultured for 5 days in E6 supplemented with FGF9 and Heparin, after which the organoids were cultured for 13 days in E6 supplemented with 50 ng/mL bone morphogenetic protein-7 (BMP7, R&D systems) and 10 ng/mL human epidermal growth factor (hEGF, Sigma Aldrich). Medium of organoids was refreshed every 2 days, or every 3 days during weekends.

### Bulk RNA sequencing

For RNA-Sequencing analysis, individual organoids were washed 2 times with ice-cold PBS and subsequently dissociated with 350µL of lysis buffer (RLT Buffer, QIAGEN). Samples were stored at -80 °C until they were delivered to USEQ Utrecht Sequencing Facility (Utrecht, the Netherlands). The provided raw transcript counts were further processed with the web application IDEP version 1.12 [30] to generate all the plots shown in the results of this manuscript. The raw data (read counts) was loaded into the IDEP1.12 webtool, normalized by counts per million (CPM) and pre-processed by filtering out genes with a lower than 0.5 CMP. The raw data was then transformed for clustering using the Edge:log2 (CMP + c) method. The heatmap was generated using the hierarchical clustered analysis using Pearson as measure of distance. The differential gene expression (DEG1 and DEG2) was analyzed using the DeSeq2 method with a false discovery rate (FDR) cutoff of 0.5 and a minimum fold-change of 1.5. From the DEG1 and DEG2, the scatterplot, the pathway tree, and the pathways networks were generated. For the pathway network analysis, the absolute values of fold changes were used for the GAGE method, sorting the pathways by both KEGG and molecular function, setting the FDR cutoff at 0.5. Lastly, the KEGG data base and Pathview were used to integrate the up- and down- regulated genes within each available KEGG pathway. The access to the raw data of the RNA sequencing can be obtained upon request. For a more detailed single-cell transcriptomic analysis of the wild-type kidney organoids, we guide the reader to the manuscript of Jansen et al. 2022 [29], in which the same iPSCs lines and differentiation protocols were used.

### Compound testing

All compounds were dissolved in DMSO unless stated otherwise. Since all drugs are already in common use, their dosage for our experiments was determined based on their non-toxic limits in previous literature with similar or same in vitro models. For the cyst formation experiments, all organoids were treated at the end of the maturation protocol (d7 + 18). Organoids were pre-incubated with the corresponding inhibitors for 8h before adding culture media with a fresh solution of inhibitor and forskolin. After 24 h of treatment, organoids were imaged under brightfield for cyst formation. For counting cysts, RGB brightfield images were converted to 8-bit and circles were drawn around them. The images were blinded, and the cysts were manually counted. Forskolin was added at a concentration of 10 µM, digoxin at 500 nM, TEA (tetraethylammonium chloride) at 500 nM, TGN-20 (2-Nicotinamide-1,3,4-thiadiazole) at 10 µM, and acetamide at 100 µM. Simvastatin and pravastatin were added at a concentration of 0.5 µM while C75 was added at a final concentration of 40 µM, and the same concentrations were kept for the combination treatments. For the statins’ experiments, all organoids were treated 6 days before the end of the differentiation and maturation protocol (d7 + 12) and media with fresh compounds was replaced daily to overcome the potential fast turnover of the compounds. For fibrosis assessment, kidney organoids were incubated for 24h with TGFβ at a final concentration of 50 ng/mL. All compounds were purchased from Sigma Aldrich and reconstituted with DMSO to a stock concentration of 1mg/mL for safe and stable storage (-20C). To normalize for the different DMSO content amongst treatment conditions, all organoids were kept at 1% DMSO content, including the untreated controls.

### Immunostainings

All stainings were performed on fixed organoids (4% PFA). After completing the maturation protocol, the organoids were washed 3 times with PBS after which the organoids were fixed with 4% PFA solution (PierceTM 16% formaldehyde (w/v), methanol-free, ThermoFisher) on ice for 20 min. After incubation, PFA was removed, and organoids were washed 3 times with PBS. Next, organoids were used for stainings, or organoids were stored in 0.1% v/v formalin at 4 °C. For antibody stainings, organoids were blocked in blocking buffer (BB) containing 10% v/v donkey fetal serum (GeneTex) and 0.06% Triton-X (ThermoFisher Scientific) in 1 × PBS. Next, primary antibodies in BB were added and incubated overnight while gently shaking at 4 °C. After incubation, primary antibody solutions were removed, and organoids were washed 3 times with 1 × PBS for 10 min while gently shaking at room temperature. Subsequently, secondary antibodies were added in 0.3% v/v triton- X in 1 × PBS and incubated for 3 h while gently shaking at room temperature. Next, secondary antibody solutions were removed, and organoids were washed 3–4 times with 1 × PBS for 10 min while gently shaking at room temperature. Finally, organoids were mounted in Prolong gold containing DAPI or without DAPI (Cell Signaling Technology, Leiden, The Netherlands). Images were captured using the confocal microscope Leica TCS SP8 X (Leica Biosystems, Amsterdam, The Netherlands). Senescent probe staining was performed using the CellEventTM Senescence Green Detection Kit (InvitrogenTM) following the manufacturer’s instructions. Lipid droplets were detected using 4,4-Difluoro-1,3,5,7,8-Pentamethyl-4-Bora-3a,4a-Diaza-s- Indacene (BODIPY) probe staining as instructed by the manufacturer. After incubation, organoids were washed 3 times with 1 × PBS for 10 min while gently shaking at room temperature. Finally, organoids were mounted in Prolong gold containing DAPI. Images were captured using the confocal microscope Leica TCS SP8 X (Leica Biosystems, Amsterdam, The Netherlands).

### Intracellular pH

Mature organoids at d7 + 18 were washed once with PBS while on a Transwell insert and, subsequently, dissociated with trypsin by adding 500 µL of trypsin in each Transwell containing 4 organoids. After 5 min incubation at 37 °C, the reaction was neutralized with E6 media and the cells were collected and centrifuged at 400rcf for 3 min. Cell pellet was then resuspended in E6 media, and 50,000 cells were seeded in 96-well plates (Corning). Cells were allowed to attach and fill the wells for an average of 2–3 days at 37 °C. On the day of the experiment, culture media containing the pHrodo™ Red P35372 probe (Thermofisher) was added to the wells and the plate was introduced into the GloMax plate reader (Promega). The first 7 min absorbance was measured at 560nm every minute for baseline pH calibration, after which the media was replaced with the same media as the start of the experiment but with 10mM NaHCO_3_ added. Absorbance was then measured every minute until 20 min total experimental time. Absorbance was corrected by total protein amount (in ng) for each sample, extracted with the Pierce BCA protein extraction kit (Thermofisher). The absorbance measurement is indirectly correlated with the pH, the lower the absorbance, the higher the intracellular pH, and vice-versa.

### Swelling assay—cyst formation

Mature organoids were imaged under brightfield, after which organoids were harvested for stainings as described above. For counting cysts, RGB brightfield images were converted to 8-bit and circles were drawn around them. The images were blinded, and the cysts were manually counted.

### LC–MS based metabolomics

For metabolomics analyses, 48h before sample collection, the normal organoid culture media was substituted by glutamine-deficient media (Gibco, # 11,960,044) with added 2mM [U-^13^C]-labeled glutamine tracer (Cambridge Isotope Laboratories). We seeded 1 organoid in each transwell insert of a 12-well plate. Organoids were washed 2 times with ice-cold PBS and 500µL of ice-cold lysis buffer (methanol/acetonitrile/dH2O at a 2:2:1 ratio) was added allowing for 1 min incubation. Organoids were mechanically dissociated by pipetting; the lysate was transferred to a 1.5mL Eppendorf and put in a shaker platform at 4 °C for 20 min. The suspension was then centrifuged at 16.000 × g for 20 min at 4 °C and the supernatants containing the metabolite suspension were collected and stored at − 80 °C until LC–MS measurement was performed. All samples were sent to the Metabolism Expertise Center (Utrecht University, Utrecht, The Netherlands) for LC–MS measurement and analysis. In short, LC–MS analysis was performed on an Q-Exactive HF mass spectrometer (Thermo Scientific) coupled to a Vanquish autosampler and pump (Thermo Scientific). Metabolites were separated using a Sequant ZIC-pHILIC column (2.1 cm × 150 mm, 5 μm, guard column 2.1 cm × 20 mm, 5 μm; Merck) with elution buffers A (acetonitrile) and eluent B (20 mM (NH_4_)_2_CO_3_, 0.1% NH_4_OH in ULC/MS-grade water (Biosolve, Valkenswaard, The Netherlands)). Gradient ran from 20% eluent B to 60% eluent B in 20 min, followed by a wash step at 80% and equilibration at 20%. Flow rate was set at 150 μL/min. Analysis was performed using the TraceFinder software (ThermoFisher Scientific, Waltham, MA, USA). Metabolites were identified and quantified based on exact mass within 5 ppm and further validated by concordance with retention times of standards. Peak intensities were normalized based on total ion count. Data was further analyzed using the publicly available code from MetaboAnalyst 5.0 [31]. The raw data can be accessed in the external links of this manuscript.

### Data analysis

Every experiment was performed in at least three biological replicates, including at least 3 technical replicates each, except for the metabolomics assays. Results are shown as the mean +/− standard error of the mean (SEM). All gathered numeric data was analyzed using GraphPad 9.0 (GraphPad software, La Jolla, CA, USA). For statistical analysis, one-way ANOVA was performed followed by Tuckey post- hoc analysis unless specified otherwise. Image analysis was performed with ImageJ (ImageJ software version 2.9.0/1.53t, National Institutes of Health, Bethesda, MD, USA). For semi-quantitative analysis of fluorescent images, RGB images were converted to 8-bit. Subsequently, grey scale thresholds were adjusted to only represent true staining. Pixel area limited to the threshold was measured for stainings or for DAPI. Eventually, pixel area of stainings was divided by the pixel area of DAPI for each image. For the image analysis, every technical replicate within each biological replicate is represented individually as a dot in the representative graphs.

## Results

### Gene editing using CRISPR/Cas9 in kidney organoids results in loss of Kir5.1

**Fig. 1:**
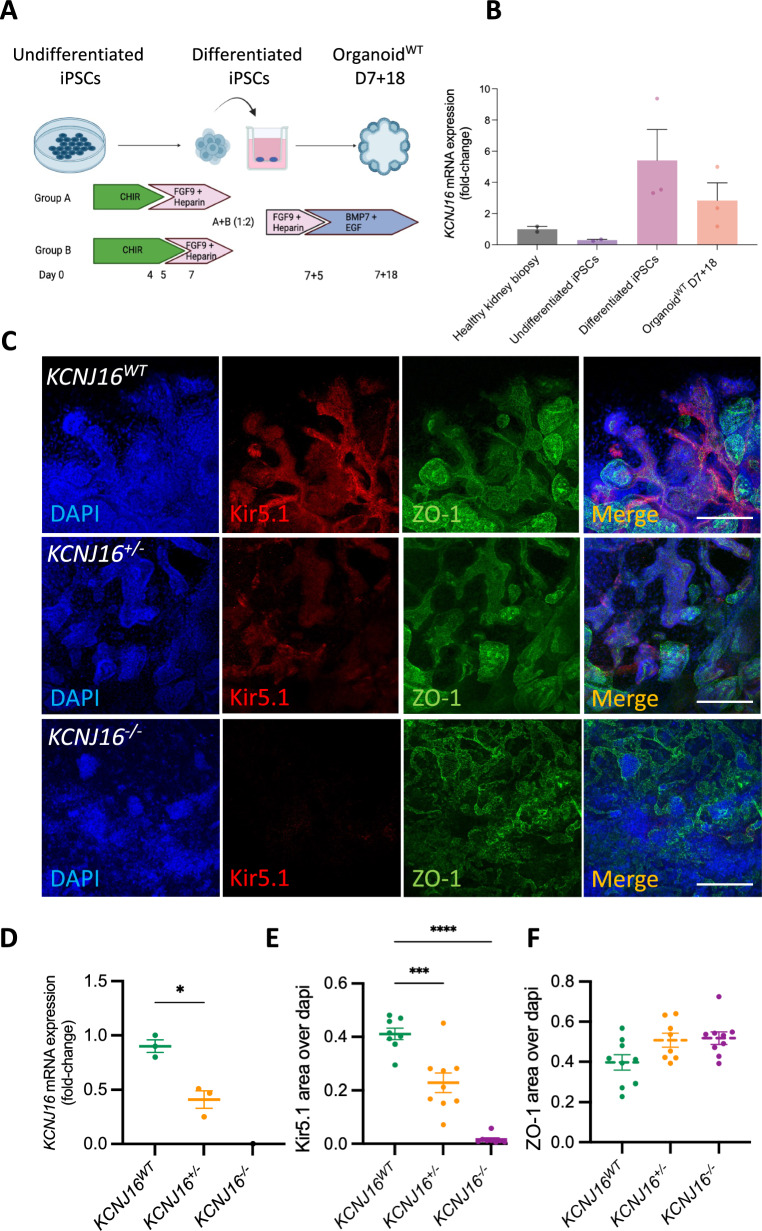
Human iPSC-derived *KCNJ16*-depleted kidney organoids show Kir5.1 depletion. **A** Schematic presentation of the differentiation protocol from undifferentiated iPSCs to mature kidney organoids. Created with BioRender.com **B** The mRNA expression levels of *KCNJ16* in undifferentiated iPSCs and mature organoids compared to the levels of a healthy human kidney biopsy sample. **C** Representative immunofluorescence images of Kir5.1 in *KCNJ16*^*WT*^, *KCNJ16*^+/−^ and *KCNJ16*^*−/−*^organoids, in conjunction with the tight-junction marker zonula occludens-1 (ZO-1). **D** Semi-quantification of the partial and total loss of Kir5.1 (*KCNJ16*) in *KCNJ16*^+/−^ and *KCNJ16*^*−/−*^ compared to *KCNJ16*^*WT*^, respectively. **E** Semi-quantification of ZO-1 staining with similar expressions in all three *KCNJ16* organoid lines. Scale bar represents 100µm. Statistics represent the significance of one-way ANOVA test (N = 3, **** = *p* < 0.0001, *** = *p* < 0.001)

To assess whether iPSC-derived kidney organoids were a suitable platform to model and study *KCNJ16* loss, the mRNA and Kir5.1 protein were measured (Fig. 1). Our results show similar mRNA expression between mature organoids and a healthy kidney biopsy (Fig. 1B), indicating their relevance as in vitro model for investigating the function of Kir5.1. Both the differentiated iPSCs and the mature kidney organoids showed higher *KCNJ16* mRNA expression (but not significant) than the healthy human kidney biopsy. The healthy kidney biopsy contains many more cell types than the kidney organoids, including epithelial cells, endothelial cells, immune cells, fibroblasts, etc. that are not present in the kidney organoids. Therefore, the mRNA expression of kidney-specific markers, such as proximal tubule markers is “diluted” in the pooled mRNA of the kidney biopsy, and we used this comparison to the healthy kidney biopsy to assess whether our kidney organoid model would be suitable for studying *KCNJ16* loss.

Next, we used CRISPR/Cas9 to generate *KCNJ16* knock-out iPSC lines. After single cell sorting, iPSC colonies were genotyped and both a clone with a homoallelic mutation (*KCNJ16*^+/−^) and a clone with a biallelic mutation (*KCNJ16*^*−/−*^) in the exon 5 of the *KCNJ16* gene were selected for further analysis (Figure S1). Immunostaining showed that Kir5.1 (*KCNJ16*) was expressed in the tubular cells in the wild type Kir5.1 (*KCNJ16*^*W*T^) (Fig. 1C), while both *KCNJ16*^+/−^ and *KCNJ16*^*−/−*^ organoids showed a partial (46%) and complete (99%) depletion of Kir5.1 expression, respectively. These results confirmed the decrease of *KCNJ16* mRNA expression in *KCNJ16*^+/−^ (60% reduction) and loss of mRNA expression in *KCNJ16*^*−/−*^ (mRNA undetectable) (Fig. 1D). The abundance of ZO-1 protein, used as reference to delineate epithelial tubular structures within the kidney organoids, seemed unaffected by the loss of Kir5.1 (Fig. 1C, F). Additionally, our transcriptomic analysis show minor differences in the expression of several key nephron segments markers upon loss of Kir5.1 (Figure S2), suggesting that development and maturation is not affected in the *KCNJ16* deficient kidney organoids.

### Loss of Kir5.1 results in acid/base homeostasis imbalance and altered ion transport

**Fig. 2:**
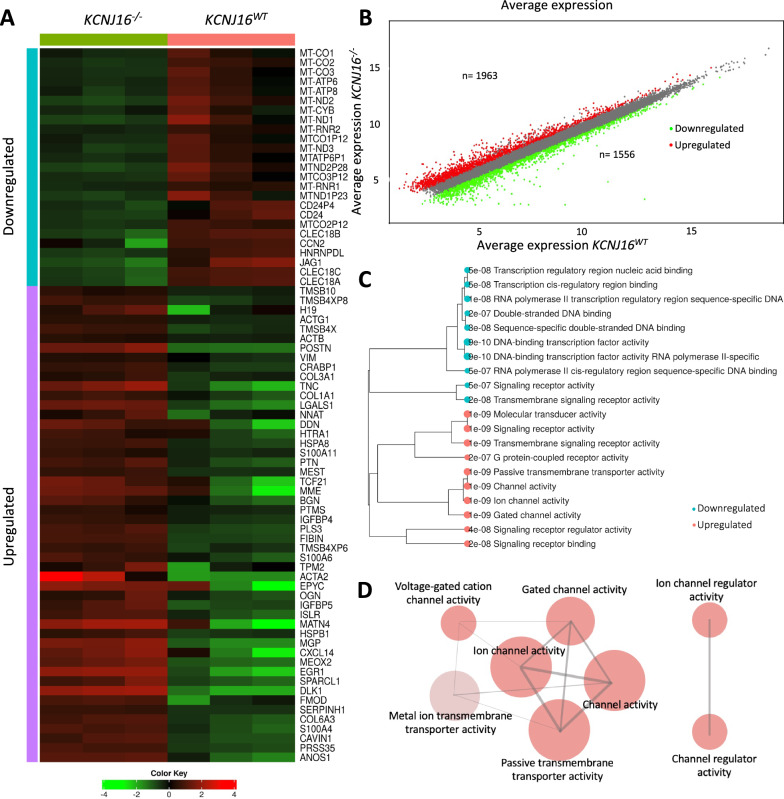
RNA-Seq analysis reveals alterations in ion- and electrolyte transporters upon loss of Kir5.1. **A** Heatmap representation of the hierarchical clustering (Pearson, p < 0.0001) including the top 100 most differentially expressed genes between *KCNJ16*^*WT*^ and *KCNJ16*^*−/−*^. **B** Scatter plot presenting the total number of up- and down- regulated genes when comparing *KCNJ16*^*−/−*^ to *KCNJ16*^*WT*^ (fold change cut off: 1.5, p < 0.0001). **C** Clustering tree presentation of the DEG2 analysis after sorting the most differentially expressed genes by molecular function in *KCNJ16*^*−/−*^. **D** Network presentation of the GAGE pathway analysis showing the most enriched pathways in *KCNJ16*^*−/−*^ sorting by molecular function (FDR cutoff: 0.5). In the network plots (**D**), the size of the nodes represents the number of genes enriching that pathway (gene set minimum size:10). The red color represents upregulated pathways, and the opacity of the nodes represents the fold enrichment (p < 0.001). Two pathways (nodes) are connected if they share at least 30% of genes. N = 3

We evaluated the transcriptional landscape of *KCNJ16*^*−/−*^ compared to *KCNJ16*^*WT*^ which revealed 1,963 upregulated and 1,556 downregulated genes (Fig. 2A,B). We clustered the significant genes by their molecular function which shows that upon loss of Kir5.1, voltage- and charge- dependent ion transporters were upregulated while DNA- and RNA-binding genes were found downregulated (Fig. 2C). Complementary, we investigated the network representation of the GAGE pathway analysis after clustering by molecular function, which confirmed that loss of Kir5.1 led to the upregulation of various cell membrane ion/cation channels and transporters dependent on charge and/or cell membrane voltage (Fig. 2D). Further exploration using KEGG pathway analysis confirmed alterations in several key transcripts involved in the cAMP pathway (Fig. S4), PT bicarbonate reclamation (Figure S5), and mineral reabsorption (Fig. S6) upon Kir5.1 loss.

**Fig. 3:**
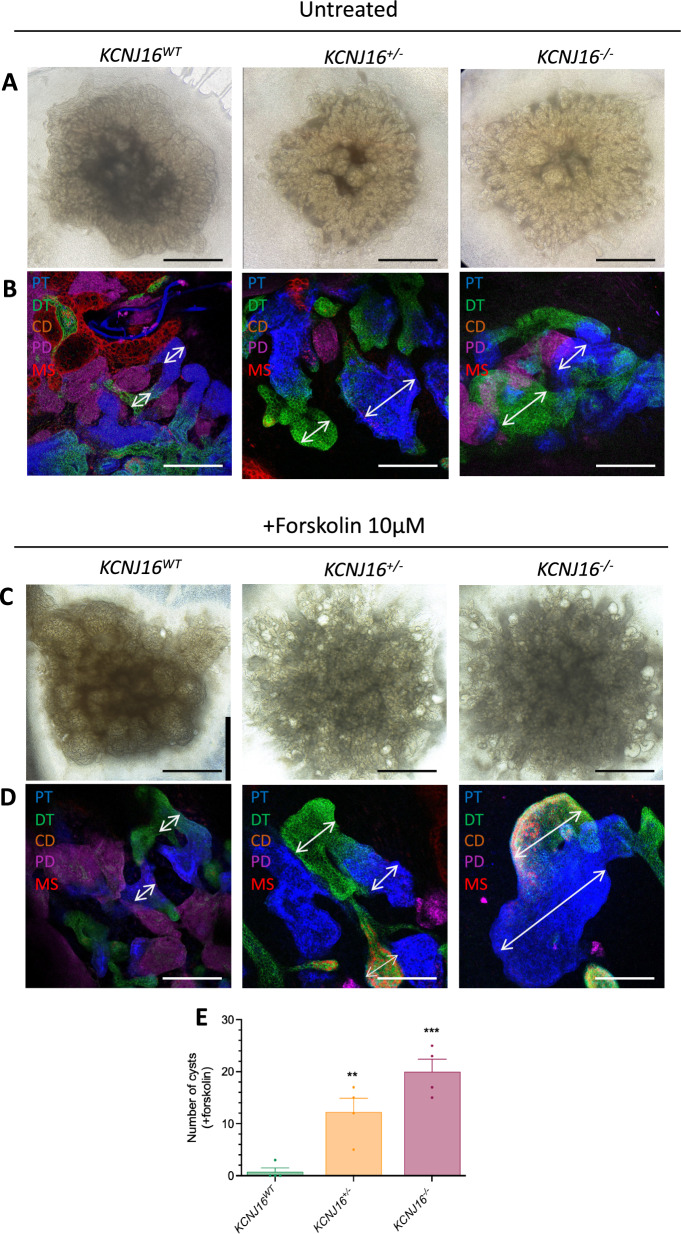
Partial and total loss of Kir5.1 results in cyst formation upon forskolin treatment. **A** Representative brightfield images of all three *KCNJ16* organoids without forskolin treatment. **B** Immunofluorescent images at of all three *KCNJ16* organoids without forskolin treatment. **C** Representative brightfield images of all three *KCNJ16* organoids after 24h treatment with 10µM forskolin. **D** Immunofluorescent images of all three *KCNJ16* organoid lines upon 24h treatment with 10µM forskolin. **E** Semi-quantification of the cyst formation. PT: proximal tubule, DT: distal tubule, CD: collecting duct, PD: podocytes, MS: mesangial cells. Scale bar represents 100µm. Statistics represent the significance of one-way ANOVA test (N = 3, *** = p < 0.001, ** = p < 0.01)

To investigate the effect of the potential ion- and electrolyte transport impairments, as well as the cAMP pathway alterations hinted by the transcriptomic data, we first evaluated the ability of the kidney organoids to respond to forskolin treatment (Figs. 3 and S3). In untreated conditions, all nephron segments could be qualitatively observed in all three lines, but we detected the presence of enlarged tubular structures (particularly proximal and distal tubules) in the *KCNJ16* and *KCNJ16*^−/−^ that were absent in *KCNJ16*^+/−^^WT^ kidney organoids (Figs. 3A, B and S3B–D). Upon treatment with forskolin, we observed the formation of cysts (mainly in the proximal and distal tubular structures) in both *KCNJ16*^+/−^ and *KCNJ16*^−/−^ (Fig. 3C, D), which were higher in number when compared to the *KCNJ16*^*WT*^ kidney organoids (Fig. 3E).

The cyst formation upon forskolin treatment indicates either a malfunction of the cAMP-PKA pathway itself, as supported by our RNA-seq data (Figure S7A), or alterations in downstream players responsible for water transport. To further investigate this, *KCNJ16*^*−/−*^ and *KCNJ16*^*WT*^ kidney organoids were treated with a combination of forskolin and an inhibitor to block either aquaporin-1 (tetra-ethylammonium; TEA), an inhibitor of urea transport (acetamide), or a compound to block Na + -K + -ATPase (digoxin) (Figure S7B-C). We observed an increase in cyst formation in the *KCNJ16*^WT^ treated with a combination of forskolin and acetamide when compared to forskolin treatment alone, while the same treatment reduced cyst formation in *KCNJ16*^−/−^ (Figure S7C). Overall, these results indicate an increased sensitivity to water imbalances upon Kir5.1 loss, especially with forskolin as stressor. Additionally, the increase in cysts formation in *KCNJ16*^WT^ together with the improvement in cysts formation in *KCNJ16*^−/−^ after adding acetamide might suggest the role of urea transport as potentially contributing to the water transport alterations.

**Fig. 4:**
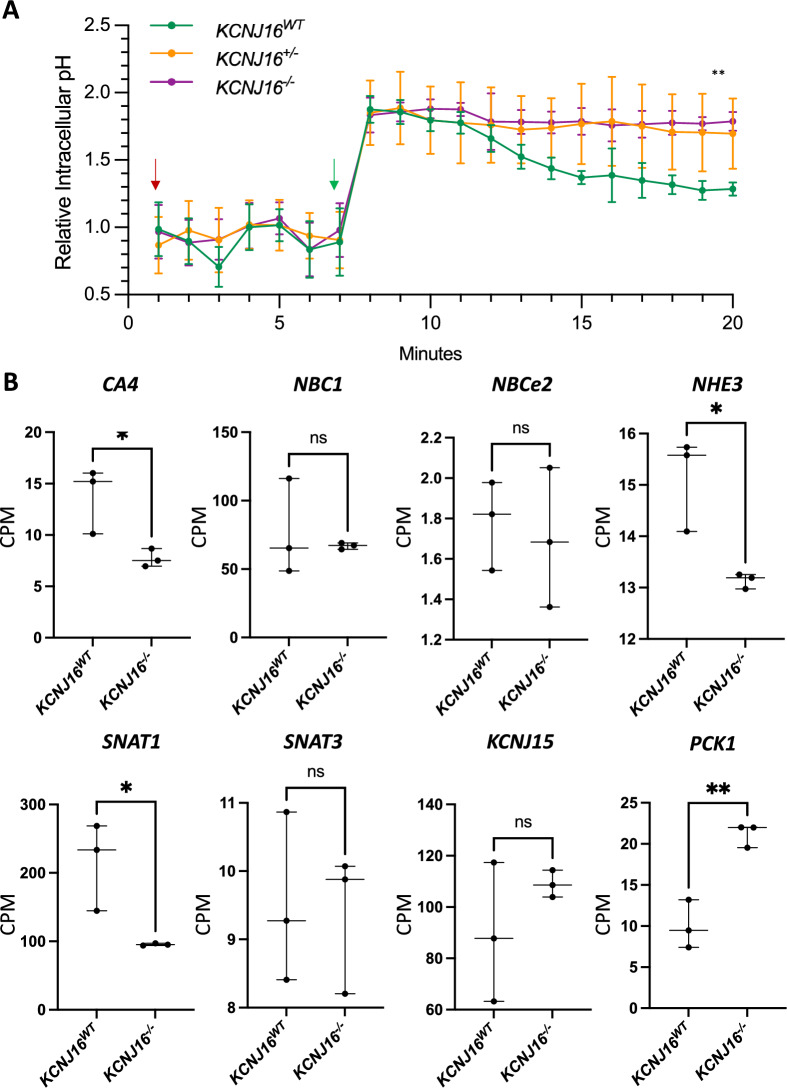
Loss of Kir5.1 results in the inability to regulate intracellular pH upon NaHCO_3_ stress. **A** Relative intracellular pH values before and after the addition of NaHCO_3_ in the culture media. The red and green arrows indicate the time of the first measurement after adding the pH probe and after adding 10 mM NaHCO_3_, respectively. **B** The mRNA levels quantified as count per million (CPM) of key genes involved in bicarbonate handling in the kidney. Statistics in panel A represent the significance of one-way ANOVA test, while statistics in panel B represent unpaired t-test (N = 3, *** = *p* < 0.001, ** = *p* < 0.01)

Secondly, we evaluated the ability of the kidney organoids to regulate intracellular pH upon stress (Fig. 4). Kidney organoids were dissociated and seeded as a monolayer. While we did not characterize the cells that remained in the 96-well plates after the organoid trypsinization and seeding, we did not observe cell detachment after seeding. Therefore, we expect that all subpopulations remained in the wells. Additionally, our results confirmed the presence of proximal tubule cells by their response to NaHCO3 uptake and intracellular pH response upon this stressor. Upon NaHCO_3_ addition, the intracellular pH of all organoid lines increased and the response of both *KCNJ16*^+/−^ and *KCNJ16*^−/−^ was comparable to that observed in *KCNJ16*^WT^ (Figure S4A). However, *KCNJ16*^−/−^ failed to restore intracellular pH after 12 min, while within that time frame *KCNJ16*^WT^ organoids partially restored their intracellular pH upon 5 min of extracellular NaHCO_3_ stress. *KCNJ16*^+/−^ organoids showed a large variability in their response to pH stress, consistent with a more intermediate phenotype. To further understand the effect of Kir5.1 loss on acid/base homeostasis, we quantified the mRNA of key transporters involved in bicarbonate handling (Fig. 4B). Both *KCNJ16*^WT^ and *KCNJ16*^−/−^ were able to increase their intracellular pH upon NaHCO_3_ addition, indicating that the transporters that allow the entry of NaHCO_3_ (such as *NBC1* and *NBCe2*) were not affected by Kir5.1 loss, while the transporters in charge of responding to a NaHCO_3_-induced acid/base stress (such as *CA4*, *SNAT1* and *SNAT3*, *NHE3*, and *PCK1*) showed altered expression levels (Fig. 4B). Furthermore, our RNA-Seq data showed an overall downregulation of key genes (including *WNK1/2/4*, *ATP1A1*, *FXYD2*, and *ENaC*) that could also play a role in acid/base homeostasis via their compensatory mechanisms upon NaHCO3 stress in the distal tubule and collecting duct (Figure S8).

### *KCNJ16* depleted kidney organoids show altered TCA cycle and lipid accumulation

**Fig. 5:**
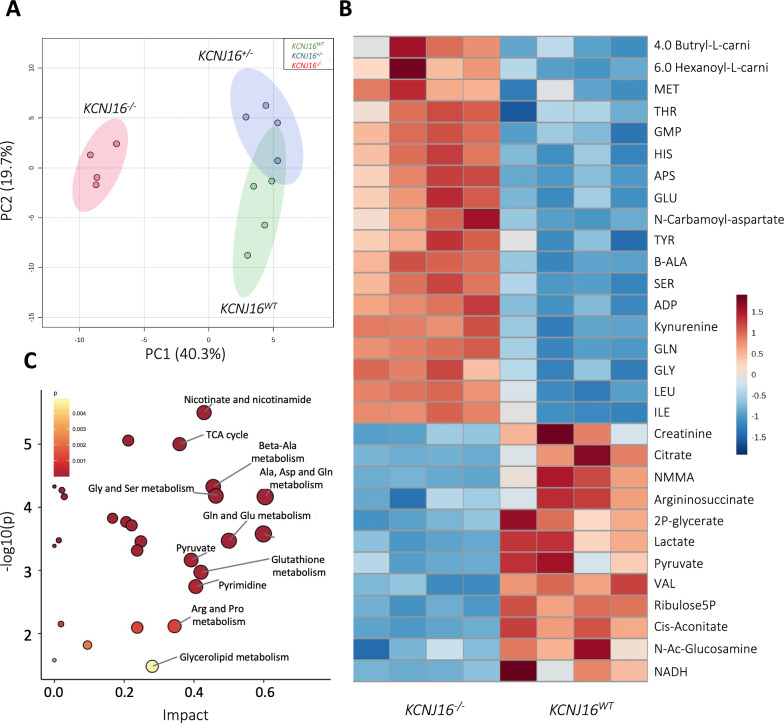
Metabolomics analysis shows the impact of the total loss of Kir5.1 on key metabolic pathways. **A** Principal component analysis (PCA) plot showing the differences between *KCNJ16*^−/−^ and both *KCNJ16*^WT^ and *KCNJ16*^+/−^, as well as the similarities between *KCNJ16*^WT^ and *KCNJ16*^+/−^, in their metabolic landscape. **B** Heatmap of the 30 most differentially expressed metabolites when comparing *KCNJ16*^WT^ and *KCNJ16*^−/−^. **C** Graphical presentation of the most differentially enriched pathways between *KCNJ16*^WT^ and *KCNJ16*^−/−^. N = 4

Next, to evaluate the downstream effect of the acid/base homeostasis disruption upon Kir5.1 loss and the ion- and electrolyte transport impairment, a metabolomic analysis was performed. While the metabolic landscape of *KCNJ16*^+/−^ and *KCNJ16*^WT^ partly overlapped, the metabolic pattern of *KCNJ16*^−/−^ clustered individually and distinct (Fig. 5A). The heatmap of differentially expressed metabolites revealed that the differences between *KCNJ16*^WT^ and *KCNJ16*^−/−^ were mostly due to differential accumulation of short chain acyl-carnitines, aminoacids, and intermediates of the TCA cycle and glycolysis (Fig. 5B). Subsequently, we found that the metabolic pathways driving the major differences between *KCNJ16*^WT^ and *KCNJ16*^−/−^ were related to amino acids, TCA cycle, and glycolipid metabolism (Fig. 5C).

**Fig. 6:**
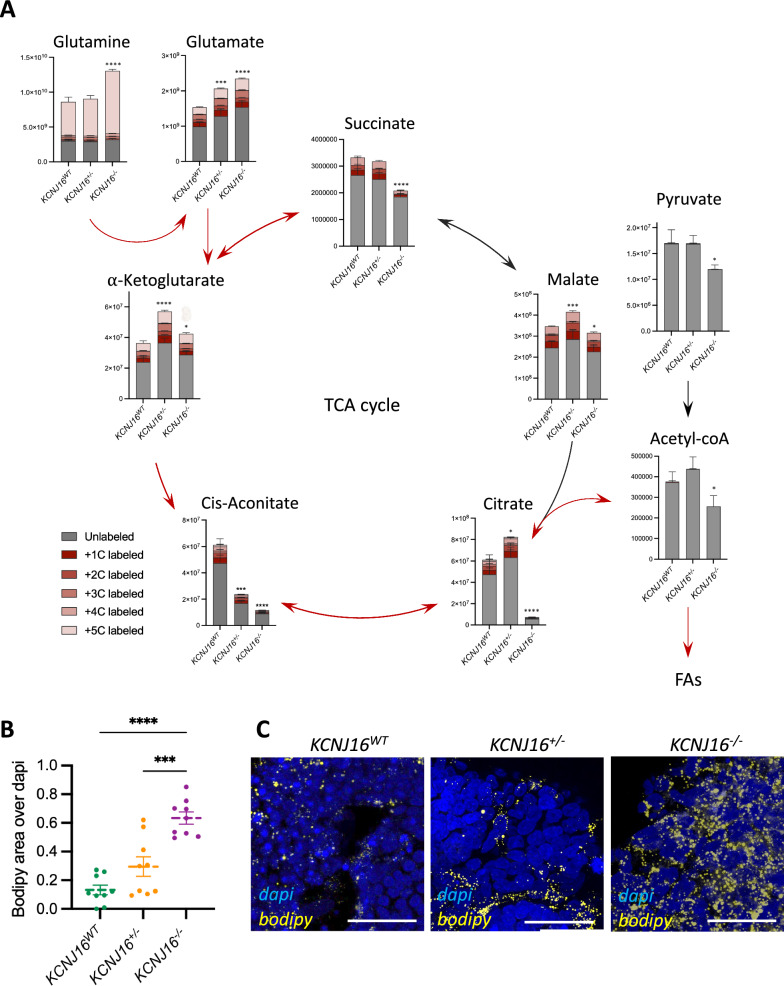
Loss of Kir5.1 results in TCA cycle impairments and lipid droplet accumulation. **A** Individual quantification of the main metabolites of the TCA cycle, including different isotopes with ^13^C (N = 4). Red arrows represent the route within the TCA cycle that results in fatty acid synthesis. **B** Semi-quantification of lipid droplet accumulation using bodipy as a probe. **C** Representative immunostainings showing the lipid droplet accumulation using bodipy as a marker (N = 3, scale bar 10µm). Statistics represent the significance of one-way ANOVA test (**** = p < 0.0001, *** = p < 0.001, * = p < 0.05) when compared to the *KCNJ16*^WT^ control values

**Fig. 7:**
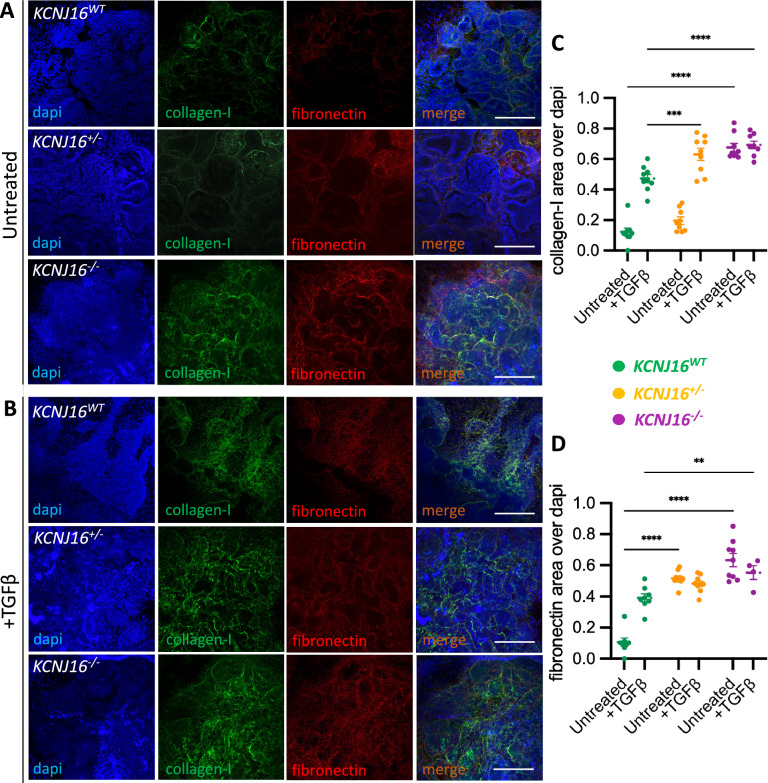
Loss of Kir5.1 results in an increased fibrotic matrix deposition. **A** Representative immunofluorescence images depicting the presence of collagen-I and fibronectin in untreated *KCNJ16*^*WT*^, *KCNJ16*^+/−^ and *KCNJ16*^−/−^ kidney organoids. **B** Representative immunofluorescence images depicting the presence of collagen-I and fibronectin in *KCNJ16*^*WT*^, *KCNJ16* and *KCNJ16*^−/−^ kidney organoids after 24h of TGFβ (50ng/mL) treatment. **C** Image quantification of collagen-I in *KCNJ16*^+/−^^*WT*^, *KCNJ16*^+/−^ and *KCNJ16*^−/−^ kidney organoids untreated and treated with TGFβ for 24h. **D** Image quantification of fibronectin in *KCNJ16*^*WT*^, *KCNJ16*^+/−^ and *KCNJ16*^−/−^ kidney organoids untreated and treated with TGFβ for 24h. Scale bar 100µm. Statistics represent the significance of one-way ANOVA tests (N = 3, **** = p < 0.0001, *** = p < 0.001, ** = p < 0.01)

To further explore the suggested TCA cycle dysfunction, we performed a metabolic flux study using ^13^C-labeled glutamine to follow the incorporation of glutamine-derived carbons in TCA cycle intermediates (Fig. 6). In terms of carbon-labelled metabolites, the relative ^13^C-labeling indicated the accumulation of glutamine and glutamate in the *KCNJ16*^−/−^ kidney organoids but relevant differences were not found in glutamine usage within the TCA cycle. Total quantification of the TCA cycle intermediates showed a slight accumulation of α-ketoglutarate (α-KG) and a generalized downregulation of the other metabolites, especially cis-aconitate and citrate in *KCNJ16*^−/−^ kidney organoids, indicating low TCA cycle activity. Additionally, *KCNJ16*^−/−^ organoids showed a reduction in pyruvate and acetyl-CoA, which together with the low levels of citrate might be indicative of the usage of the TCA intermediates towards fatty acid synthesis. To evaluate this, we analyzed the presence of lipid droplets in all three kidney organoid lines (Fig. 6). *KCNJ16*^−/−^ organoids clearly demonstrate accumulation of lipid droplets as compared to *KCNJ16*^WT^, while the heterozygous organoids show an intermediate phenotype (Fig. 6B, C). The accumulation of lipids was accompanied by an increased deposition of extracellular matrix proteins, as evaluated by collagen-I and fibronectin (Fig. 7). Our results show that without external stimulation, *KCNJ16*^−/−^ organoids accumulated collagen-I and fibronectin compared to the untreated *KCNJ16*^WT^, while *KCNJ16*^+/−^ organoids only showed increased collagen-I when stimulated with TGFβ (Fig. 7). These results indicate that total Kir5.1 loss promotes fibrosis while having a functional KCNJ16 allele shows an ameliorated phenotype.

### Statins prevent lipid accumulation in *KCNJ16*-depleted kidney organoids

**Fig. 8:**
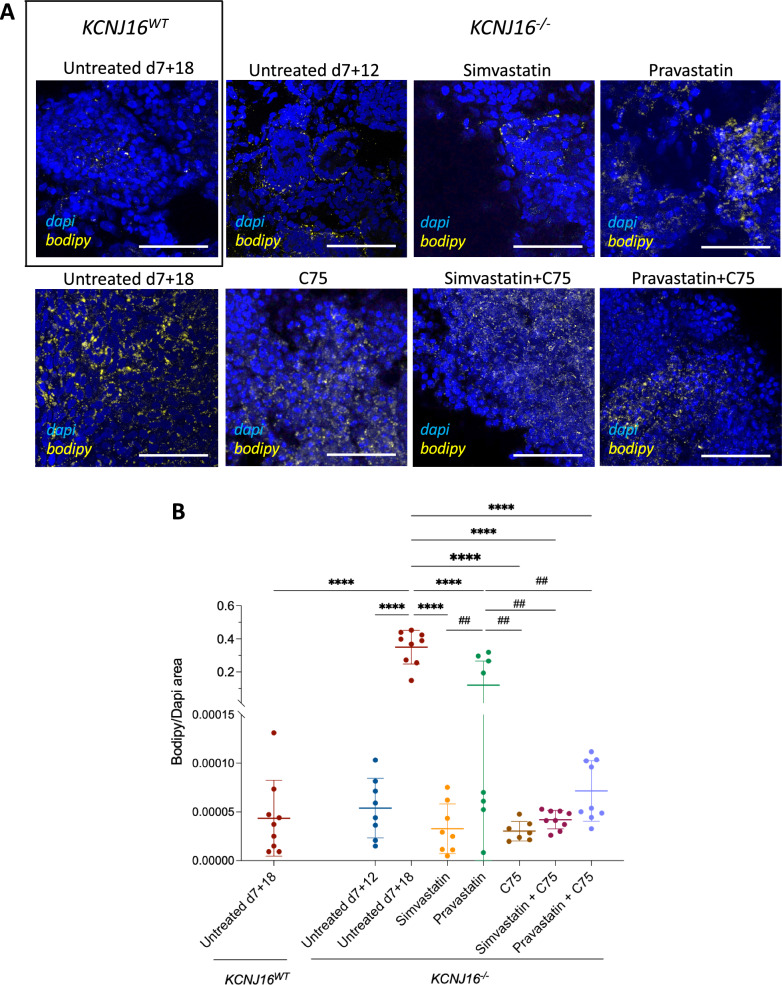
Treatment with statins prevents lipid droplet accumulation in *KCNJ16*^−/−^ kidney organoids. **A** Representative immunofluorescence images of lipid droplets in *KCNJ16*^−/−^ organoids after treatment with Simvastatin, pravastatin, C75, and combinations. All compound treatments were administered at d7 + 12 and refreshed daily until d7 + 18. **B** Semi-quantification of droplet accumulation imaging data. Simvastatin and pravastatin were used at a final concentration of 0.5 µM and C75 was used at a concentration of 40 µM. Scale bar 100µm. Statistics represent the significance of one-way ANOVA tests (N = 3, **** = p < 0.0001 when comparing all conditions to the untreated *KCNJ16*^−/−^ at d7 + 18, and ^##^ = p < 0.01 when comparing treatments amongst each other)

Finally, we investigated whether the lipid droplet accumulation and matrix proteins deposition could be reversed by treating *KCNJ16*^−/−^ organoids with the statins simvastatin or pravastatin, or a fatty acid synthase blocker (C75), alone and in combinations (Figs. 8 and S10). Since we observed that lipid droplets started to accumulate in *KCNJ16*^−/−^ within the last 6 days of maturation, organoids were exposed daily to the drugs starting from d7 + 12. Statins and C75 all effectively reduced lipid droplet accumulation in *KCNJ16*^−/−^ organoids to matured, untreated *KCNJ16*^WT^ organoid levels (Fig. 8 A-B). Furthermore, statins and C75 reduced collagen I deposition alone and in combination, with the two combination treatments being most effective (Figure S9). In contrast, we observed no differences for fibronectin deposition under the same treatment conditions. These findings suggest that statins and a fatty acid synthase blocker could prevent the lipid accumulation and ameliorate the collagen-I deposition associated with Kir5.1 deficiency.

## Discussion

In this study, we aimed to generate and characterize a novel *KCNJ16* knock-out kidney organoid model to provide an advanced and robust platform to further study this recently described kidney tubulopathy [2]. Despite our kidney organoid models are not compatible with direct measuring of intracellular potassium, our results show that total loss of Kir5.1 (*KCNJ16*) led to transcriptomic impairment of key transporters regulating essential cellular homeostasis and metabolic processes, which resulted in pH imbalance, cyst formation and metabolic landscape impairment, with particular impact on the TCA cycle and lipid metabolism. Subsequently, the depletion of Kir5.1 resulted in lipid droplet accumulation and fibrosis, which was successfully prevented by treatment with statins.

Supporting evidence of the effects of only Kir5.1 depletion remains limited because of a lack of robust in vitro and in vivo models and limited clinical cases. Our results show that upon Kir5.1 depletion, the transcriptomic level (Figs. 4, S4 and S5) and activity of ion, electrolyte, salt, and water transporters is impaired, reducing the ability of *KCNJ16*^−/−^ organoids to regulate their water transport and intracellular pH upon external stimuli. Membrane potential and pH imbalances have been directly linked to electrolyte homeostasis and water balance impairment, especially in the kidney [32–34], which could potentially lead to water accumulation and cyst formation, as observed in our *KCNJ16*^−/−^ kidney organoids. Although the specific role of Kir5.1 is yet unknown, our results show that loss of function of *KCNJ16* may alter the same feedback loop between water/electrolyte transport and pH homeostasis which could eventually result in the phenotype observed in patients, including salt wasting, hypokalemia and metabolic acisosis [2]. To further understand the inability to regulate water transport upon forskolin stress, we pharmacologically inhibited the transport of sodium, potassium, water, bicarbonate and urea, and suggested that urea transport to might be altered upon Kir5.1. However, these results must be taken with caution because of a lack of specificity of the inhibitors used [35–38].

Another key feature identified upon Kir5.1 loss is the impairment in the TCA cycle and lipid metabolism. The defects we detected in the TCA cycle could be due to changes in the activity of enzymes that regulate either the TCA cycle itself or that are involved in the synthesis and processing of the TCA cycle precursors, such as citrate synthase, aconitase and pyruvate dehydrogenase. The impaired functioning of these enzymes could directly lead to disruptions in glucose and glutamine metabolism, which could in turn affect the functioning of the TCA cycle by limiting the presence of key sources. Our transcriptomic data did not show significant differences in these enzymes; however, their activity might still be impacted by alterations in the acid/base environment they are in [39–42]. Furthermore, potential changes in activity of enzymes that regulate the amino acids and lipid metabolism could either further contribute to the observed impairments in metabolic pathways or be a consequence of it, given that these pathways directionality change depends on the energy demands of the cells. Citrate showed to be the most differentially expressed metabolite within the TCA cycle when comparing *KCNJ16*^WT^ and *KCNJ16*^−/−^ organoids. This metabolite is involved in several metabolic pathways [43, 44] and its observed loss in *KCNJ16*^−/−^ could have different implications in pathways in addition to the TCA cycle [45]. Citrate can be either processed further within the TCA cycle directly into succinate, but it can also enter a different loop outside the TCA cycle towards lipid synthesis, depending on the energy demands and the anabolic/catabolic status of the cells [46–48]. Our results lead us to hypothesize that citrate exits the TCA cycle to enter the lipid biosynthesis pathway, a phenomenon that has been observed in multiple diseases, including cancer [49] and diabetic nephropathy [50]. Furthermore, it has been shown previously that low conversion of phosphoenolpyruvate into pyruvate can lead to the accumulation of glycolytic intermediates [51], which can then be used for either nucleotide synthesis and/or lipid synthesis [52]. Although we observed reduced levels of pyruvate in *KCNJ16*^−/−^, our results showed lower levels of glycolytic intermediates as well as a lower presence of TCA intermediates leading towards fatty acid synthesis, such as citrate and acetyl-CoA. Altogether, this might be indicative of an overall lower glycolytic activity in the *KCNJ16*^−/−^ kidney organoids.

It is known that kidney epithelial cells have high energy requirements, which are largely met by fatty acid oxidation. Additionally, complex changes in lipid metabolism are observed in patients with kidney disease. Defects in fatty acid oxidation and increased lipid uptake, especially in the context of hyperlipidemia and proteinuria, contribute to this excess lipid build-up and exacerbate kidney disease progression. In the context of kidney organoids, it has been observed that there is a dominant metabolic alteration from glycolysis to oxidative phosphorylation during the differentiation process. This shift is crucial for the organoids to mature and function properly [53]. Specifically, glycine, serine, and threonine metabolism have been shown to play a regulatory role during kidney organoid formation and lineage maturation. Considering this, the metabolic deficiencies in *KCNJ16*-depleted kidney organoids due to the loss of Kir5.1 could potentially impact kidney organoid formation and nephron segment maturation. We assessed the mRNA expression of key markers for each segment of the nephron (Fig. S2) as well as provided evidence of immunostainings for the four main nephron subpopulations (Fig. S3). Our results in Figures S2 and S3 show no significant differences in expression levels and nephron structures upon Kir5.1 loss when compared to the wild-type.

Lipid droplet aggregation in the kidney and consequent sustained lypotoxicity have been correlated with kidney fibrosis and eventually chronic kidney disease [46, 54–56]. Despite kidney fibrosis has, as of yet, not been described as a phenotype for *KCNJ16-* related kidney disease, we observed an increase in the fibrotic markers collagen-I and fibronectin in our *KCNJ16*-depleted kidney organoids when compared to the *KCNJ16*^WT^ control. We hypothesize that kidney fibrosis might appear because of our model itself as our in vitro system does not allow for rescue mechanisms to detect or halt the potential damage derived from Kir5.1 loss, and the culture conditions have not been optimized for the need of these cells. Numerous studies have shown that restoring either the defective fatty acid degradation or fatty acid synthesis in kidney cells can mitigate kidney fibrosis progression [57–60]. Statins have gained popularity in pharmacological research because of their high efficacy to reduce lipid accumulation and preventing disease progression originated from lipid disorders, such as diabetic nephropathy and chronic kidney disease [61–63]. Our results showed that 6-days treatment with non-toxic doses of fatty acid synthase inhibitors, especially the combination of simvastatin and C75, was able to inhibit lipid droplet accumulation and subsequently reduce collagen-I deposition. Although exhibiting normal urinary concentrating ability, these patients manifest diverse health complications, including metabolic alkalosis/acidosis, hypokalemia, salt wasting, and sensorineural deafness, significantly influencing their quality of life [2]. Consequently, the potential amelioration or suppression of these symptoms through statins treatment holds considerable importance for both the affected individuals harboring Kir5.1 defects and the broader domain of other lipid-related kidney conditions.

Altogether, these findings confirm that the loss of both healthy alleles of the *KCNJ16* gene negatively impacts and changes the metabolic landscape of kidney organoids towards TCA cycle alterations and induces lipid droplet accumulation. While only biallelic mutations in the *KCNJ16* gene appeared disease causing, we included the *KCNJ16*^+/−^ clone to investigate whether monoallelic mutations in *KCNJ16* may express a tubulopathy phenotype as well, which could provide insight into whether carriers of *KCNJ16* mutations are at risk of developing kidney disease. The *KCNJ16*^+/−^ organoids showed a high variability response to changes in pH and overall metabolism was similar to *KCNJ16*^WT^, confirming that only biallelic mutations cause tubulopathy while having one healthy allele remaining seems to show an ameliorated version, still comparable to the wild-type phenotype.

## Conclusions and future directions

In this study, we generated a novel kidney organoid model using CRISPR/Cas9 technology to investigate the recently described *KCNJ16* kidney tubulopathy, confirming the functional impact of *KCNJ16* mutations and revealing metabolic impairment and lipid droplet accumulation in the compound heterozygous mutated organoids. Our findings highlight the importance of kidney organoids and CRISPR/Cas9 technology for the in vitro validation of candidate genes, further studying disease phenotypes, and evaluating potential treatment options. While our model provides a valuable tool for understanding the disease phenotype and aid to find therapeutic strategies, future directions should focus on validating these findings in in vivo models to further explore lipid accumulation as a novel potential marker for disease detection and to evaluate further interventions to improve patient outcomes. Conjointly, these efforts will contribute to advancing our understanding of *KCNJ16* related kidney disease and aid in the development of personalized approaches for diagnosis and treatment. This approach opens the possibility of developing advanced in vitro models for other rare and underrepresented genetic kidney diseases, for which patient material and effective treatments are limited.

### Supplementary Information

Additional file 1. Figure S1. Schematic representation of the generation of KCNJ16 knockouts in iPSCs. The iPSCs KCNJ16WT were nucleofected with a gRNA targeting a genomic region of exon 5 of the KCNJ16 gene with an average knockout efficiency score on the pooled population of 75% (N=3). After transfection, cells were single-cell sorted and expanded until DNA collection was possible. Out of 120 clones sorted, only 26 grew to colonies. Not all clones recovered were genotyped, sequencing of the clones was stopped after obtaining a KCNJ16+/- and a KCNJ16-/- clones in which mutations were predicted to be deletereous framshifts. Only one clone out of the 10 sequenced was not harboring any mutation in the target site, therefore the targeting percentage is an estimated 90%. Created with BioRender.com. Figure S2. Total loss of Kir5.1 does not impact nephron markers expression. Heatmap depicting the minor mRNA expression differences (Z-score between -0.7 and +0.7) when comparing the mRNA expression several markers for each of the four major nephron segments present in the kidney organoids. Figure S3. Extended data depicting the brightfield and coupled nephron segments immunostaining images of untreated and 10µM forskolin treated kidney organoids. Under untreated conditions (Panel A-B), the organoids showed several tubular structures under the brightfield (Panel A), which was then confirmed by the presence of all nephron segments with immunofluorescence (Panel B), including proximal tubule and distal tubule structures (in blue and green, respectively). These tubular structures were found enlarged upon treatment with 10µM forskolin, which we detected by imaging under brightfield (Panel C) as well as with immunofluorescence (Panel D). While the tubular structures might not be too straightforward to detect under the brightfield, we confirmed their presence with the complementary immunostaining panels. For this assay, 3 biological replicates (3 kidney organoids) were harvested, blinded and analyzed for cyst count. Figure S4. Overview of differentially expressed genes involved in the cAMP signaling pathway. Figure S5. Overview of up- and down- regulated genes involved in the PT bicarbonate reclamation. Figure S6. Overview of up- and down- regulated genes involved in mineral reabsorption in the PT. Figure S7. Loss of Kir5.1 results in electrolyte and ion transport impairment. Figure S8. The mRNA levels (CMP) of key genes related to ion and salt transport in the distal part of the nephron. Figure S9. Treatment with statins prevents fibrotic fibers deposition in KCNJ16-/- kidney organoids (PDF 8698 kb)

## Data Availability

The datasets generated during the current study in regard to the bulk RNA-Sequencing and Metabolomics are available in the figshare repository. Metabolomics data: [https://figshare.com/articles/dataset/KCNJ16_Metabolomics_data_All_raw_folders/25702710]. Bulk RNA-Sequencing data: [https://figshare.com/articles/dataset/JAN7448_processed_exon_featureCounts_raw_KCNJ16_txt/25663905]. Other datasets used and/or analyzed during the current study are available from the corresponding author on reasonable request.

## Abbreviations

α-KG: α-Ketoglutarate
BB: Blocking buffer
CD: Collecting duct
CPM: Counts per million
DCT: Distal convoluted tubule
DEG1: Differential gene expression 1
DEG2: Differential gene expression 2
FACS: Fluorescence-activated cell sorting
FDR: False discovery rate
IMAGEN: IMplementation of Advancements in GENetic Kidney Disease
iPSCs: Induced pluripotent stem cells
KCNJ16: Potassium Channel, Inwardly Rectifying Subfamily J Member 16
NWO: Nederlandse organisatie voor Wetenschappelijk Onderzoek (Netherlands Research Council)
PBS: Phosphate buffered saline
PFA: Paraformaldehyde
PT: Proximal Tubule
RNP: Ribonucleoprotein
SCTC: Stem Cell Technology and differentiation Center
SEM: Standard error of the mean
TEA: Tetraethylammonium chloride
TGN-20: 2-Nicotinamide-1,3,4-thiadiazole
