# Supplementary figures and images for: KCNJ16-depleted kidney organoids recapitulate tubulopathy and lipid recovery upon statins treatment
Source: Stem Cell Res Ther. 2024 Aug 26;15:268. doi: 10.1186/s13287-024-03881-3 (PMC11346019; doi:10.1186/s13287-024-03881-3)

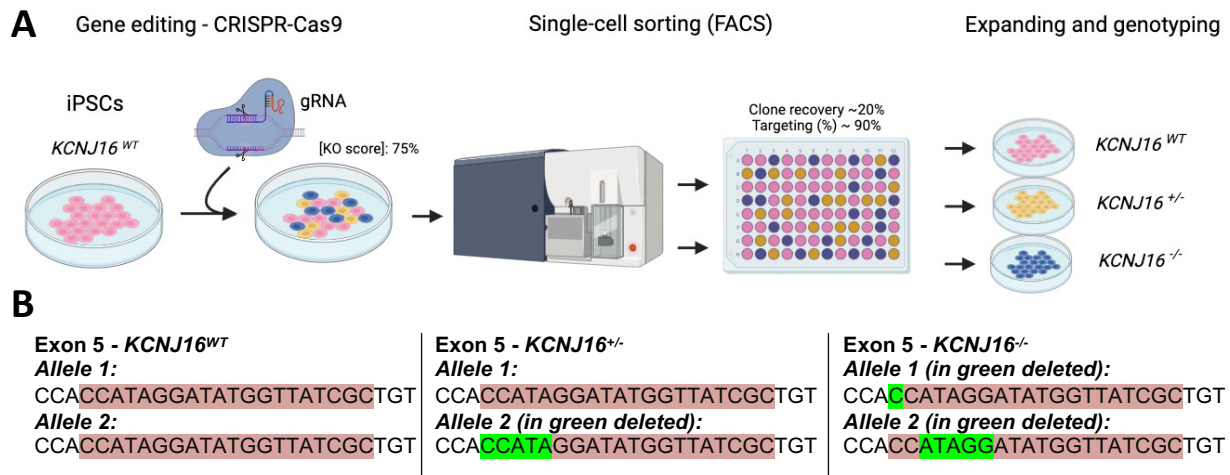

Figure S1

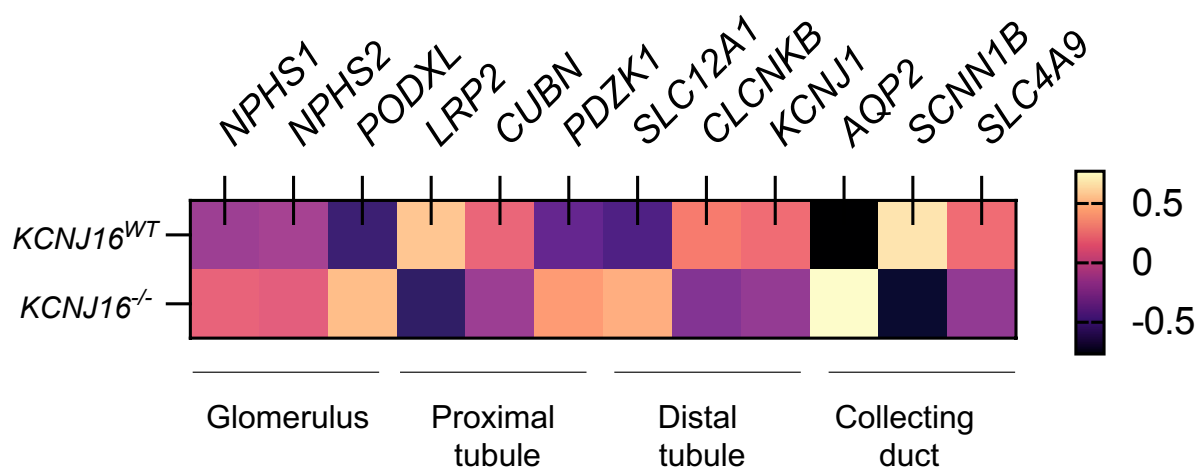

Figure S2

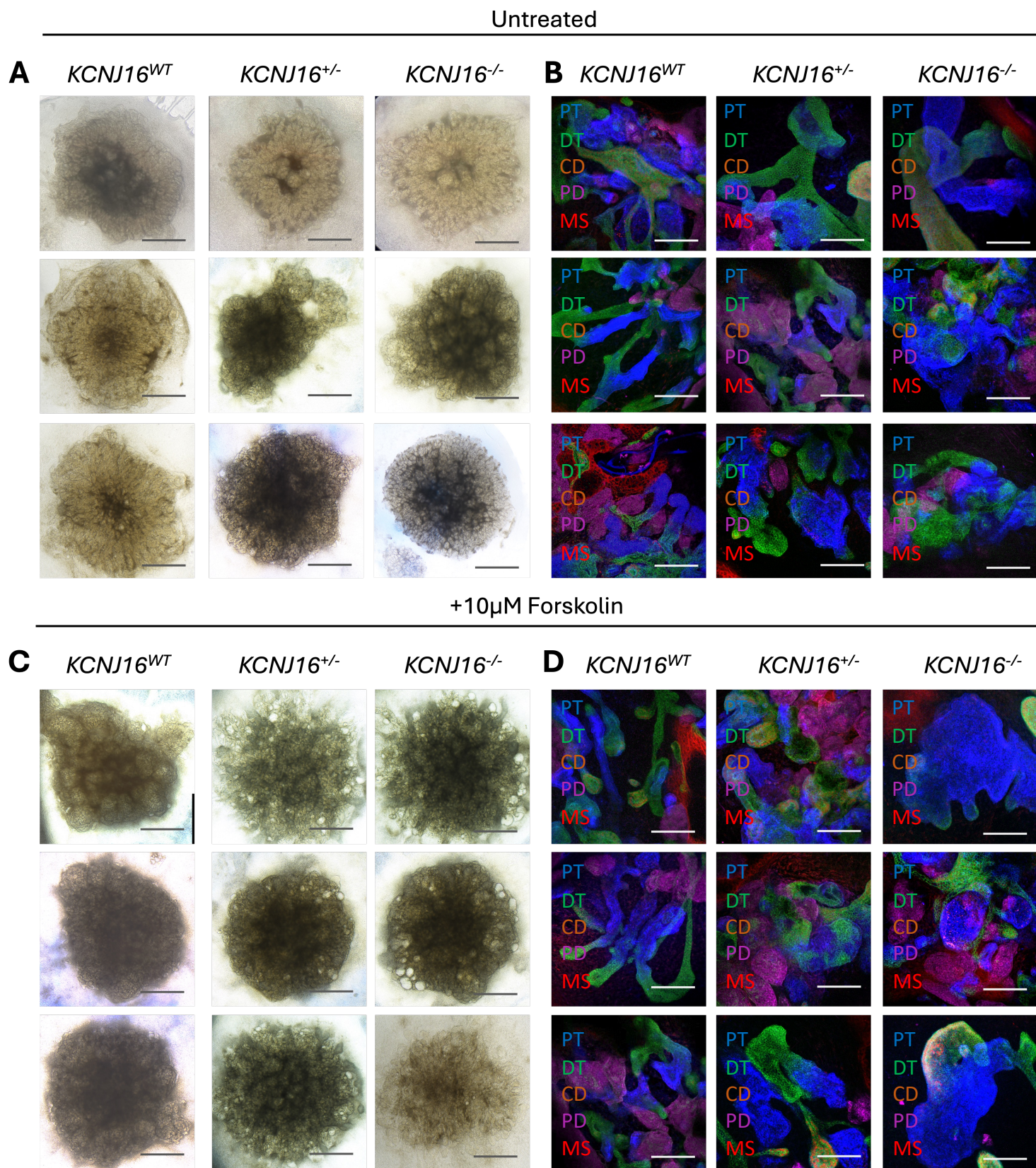

**Figure S3**

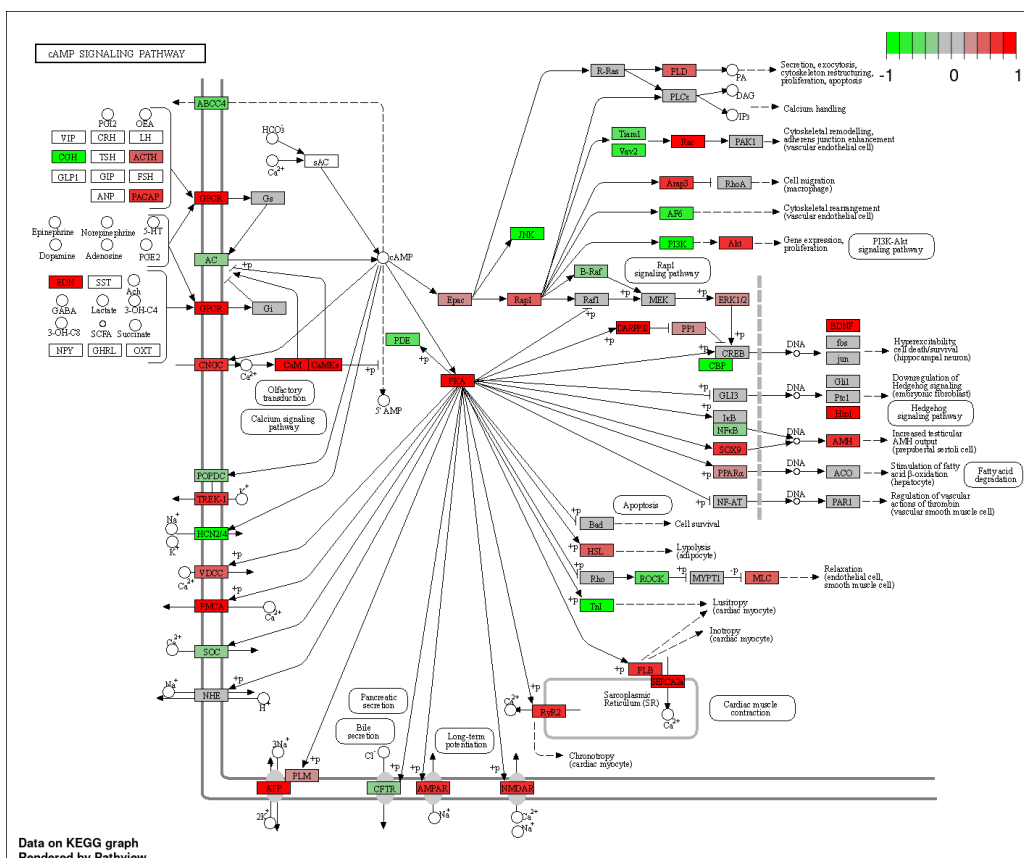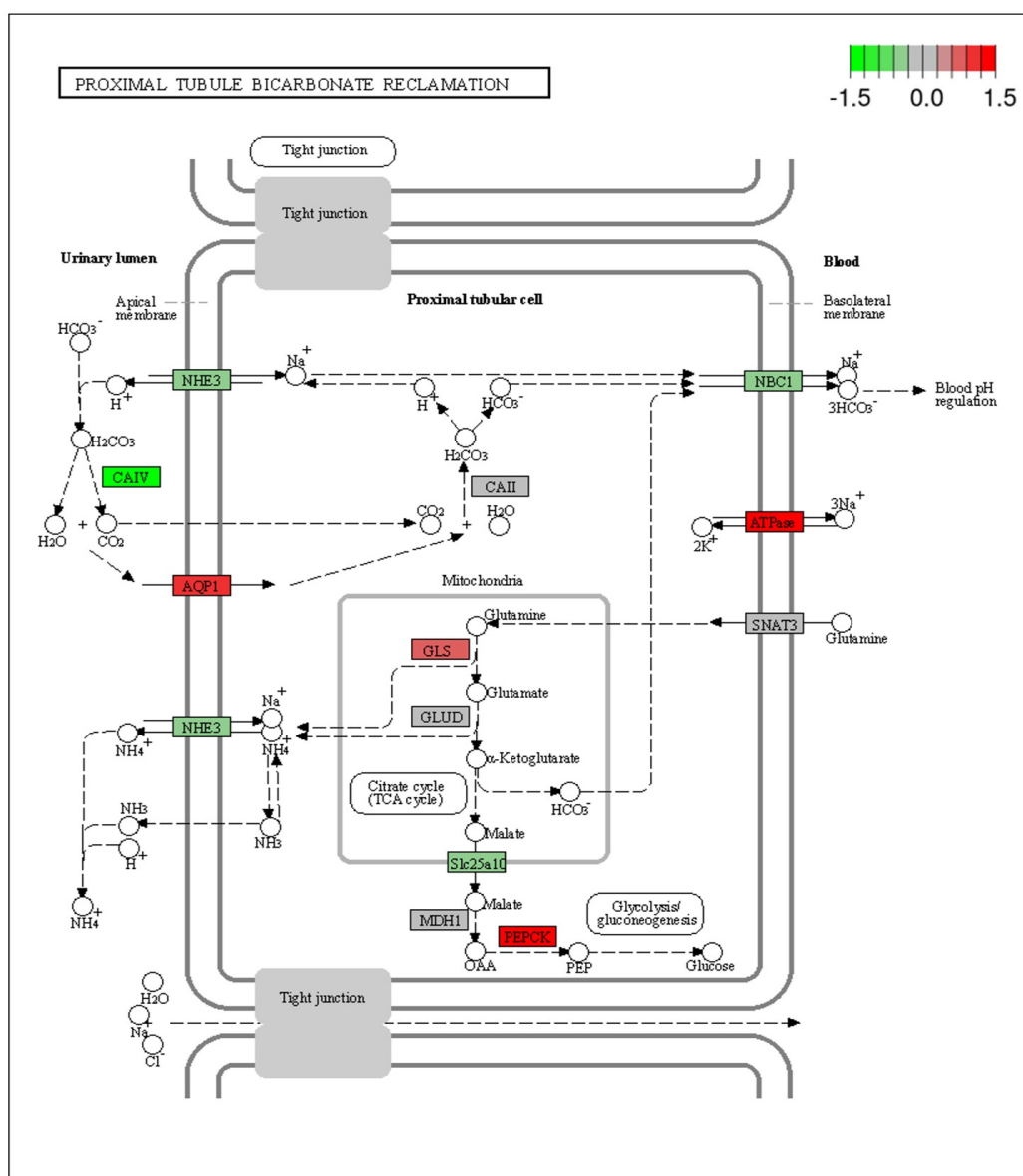

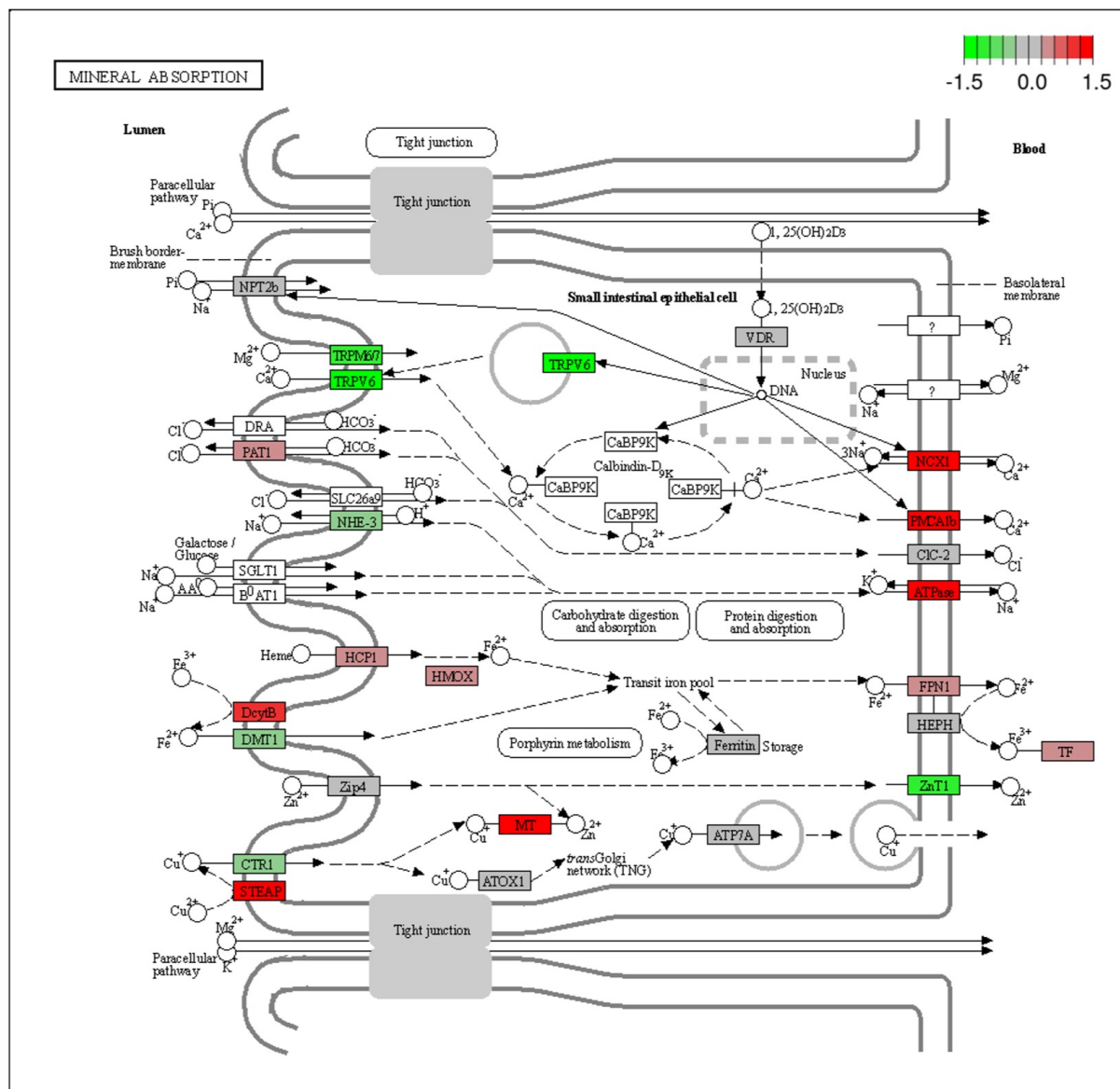

Figure S6

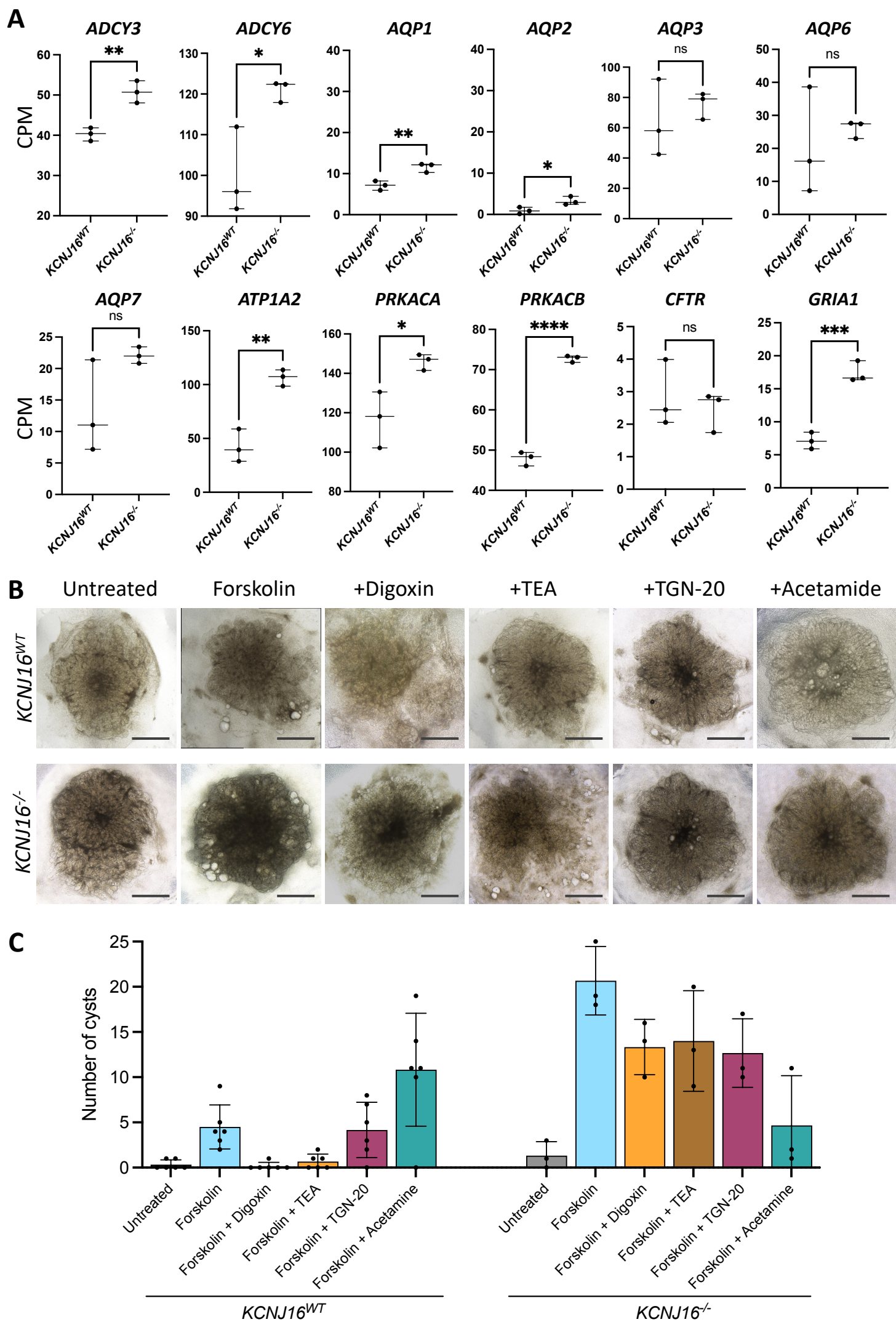

Figure S7

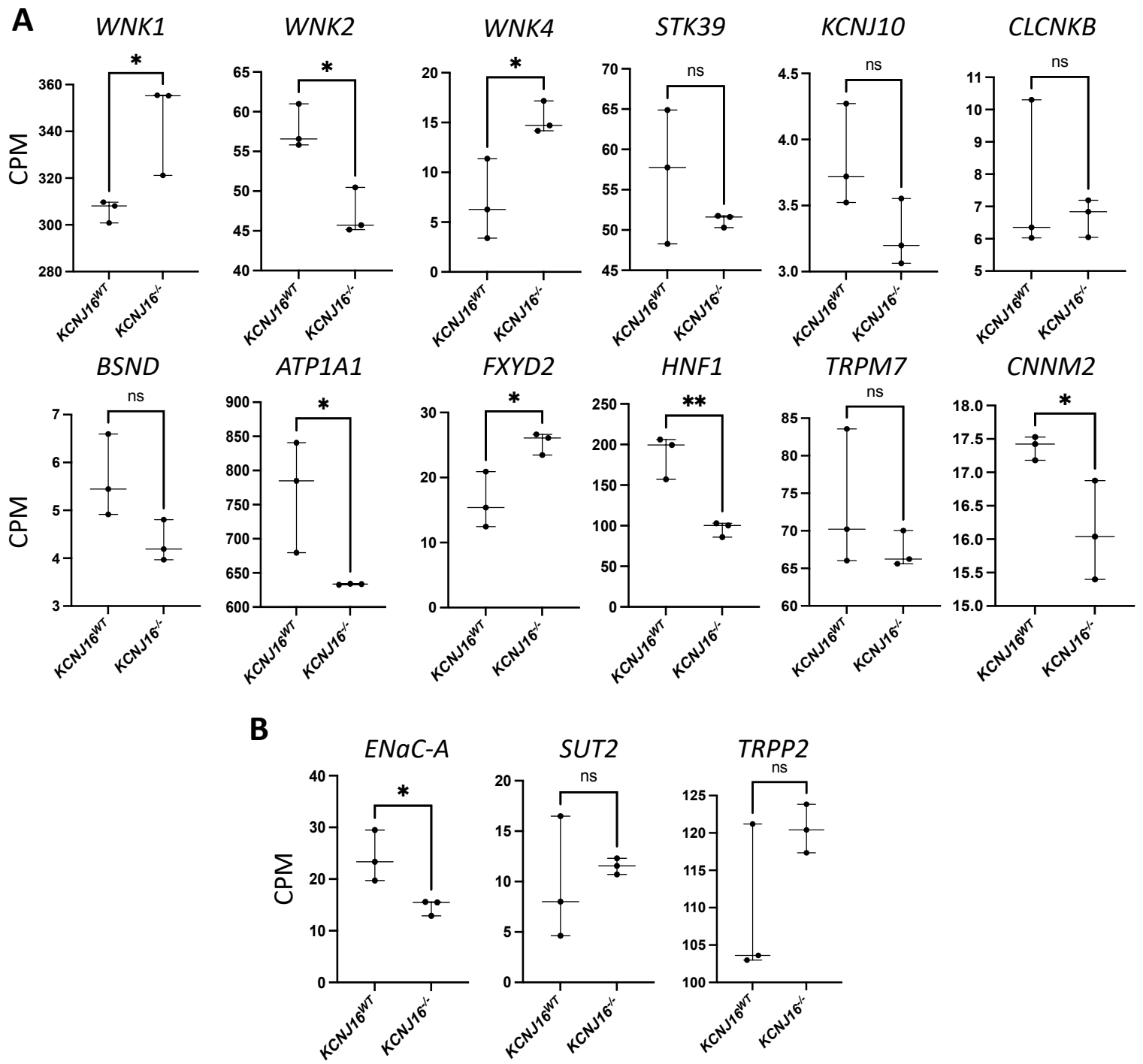

Figure S8

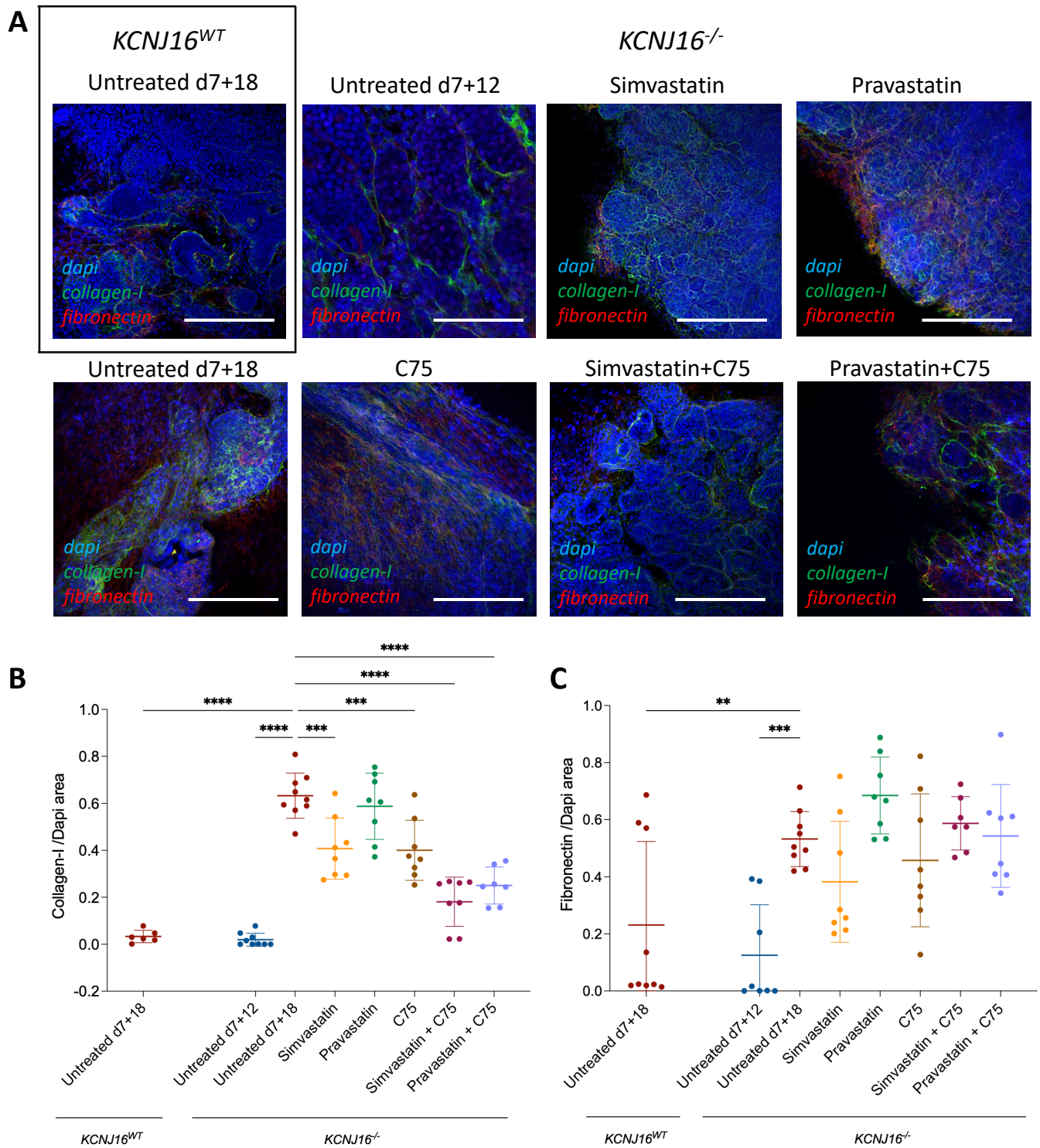

Figure S9

Supplement: Supplementary file 1 — Additional file 1. Figure S1. Schematic representation of the generation of KCNJ16 knockouts in iPSCs. The iPSCs KCNJ16WT were nucleofected with a gRNA targeting a genomic region of exon 5 of the KCNJ16 gene with an average knockout efficiency score on the pooled population of 75% (N=3). After transfection, cells were single-cell sorted and expanded until DNA collection was possible. Out of 120 clones sorted, only 26 grew to colonies. Not all clones recovered were genotyped, sequencing of the clones was stopped after obtaining a KCNJ16+/- and a KCNJ16-/- clones in which mutations were predicted to be deletereous framshifts. Only one clone out of the 10 sequenced was not harboring any mutation in the target site, therefore the targeting percentage is an estimated 90%. Created with BioRender.com. Figure S2. Total loss of Kir5.1 does not impact nephron markers expression. Heatmap depicting the minor mRNA expression differences (Z-score between -0.7 and +0.7) when comparing the mRNA expression several markers for each of the four major nephron segments present in the kidney organoids. Figure S3. Extended data depicting the brightfield and coupled nephron segments immunostaining images of untreated and 10µM forskolin treated kidney organoids. Under untreated conditions (Panel A-B), the organoids showed several tubular structures under the brightfield (Panel A), which was then confirmed by the presence of all nephron segments with immunofluorescence (Panel B), including proximal tubule and distal tubule structures (in blue and green, respectively). These tubular structures were found enlarged upon treatment with 10µM forskolin, which we detected by imaging under brightfield (Panel C) as well as with immunofluorescence (Panel D). While the tubular structures might not be too straightforward to detect under the brightfield, we confirmed their presence with the complementary immunostaining panels. For this assay, 3 biological replicates (3 kidney organoids) were harvested, b [file 13287_2024_3881_MOESM1_ESM.pdf]
